# Association of low-voltage areas with the regional wall deformation and the left atrial shape in patients with atrial fibrillation: A proof of concept study

**DOI:** 10.1016/j.ijcha.2021.100730

**Published:** 2021-02-26

**Authors:** Sotirios Nedios, Soroosh Sanatkhani, Michael Oladosu, Timm Seewöster, Sergio Richter, Arash Arya, Jordi Heijman, Harry J.G.M. Crijns, Gerhard Hindricks, Andreas Bollmann, Prahlad G. Menon

**Affiliations:** a Heart Center, University of Leipzig, Germany; b Cardiac Arrhythmia Service, Massachusetts General Hospital, Boston, MA, USA; c Department of Cardiology and Cardiovascular Research Institute Maastricht, Maastricht University Medical Center, the Netherlands; d University of Pittsburgh, Pittsburgh, PA, USA; e Duquesne University, Pittsburgh, PA, USA; f QuantMD LLC, Pittsburgh, PA, USA

**Keywords:** Atrial fibrillation, Atrial remodeling, Computer tomography, Voltage mapping, Sphericity, ASI, asymmetry index, AF, atrial fibrillation, AR, average radius, CA, catheter ablation, CT, computed tomography, IQR, inter-quartile range, LAA, left atrial appendage, LA, left atrium, LA-A, left atrial anterior (LA-A) partial volume, LAV, left atrial volume with anterior (LA-A) and posterior (LA-P) partial volumes, LA-P, left atrial posterior (LA-P) partial volume, LVA, low-voltage area, LV, left ventricle, LVDD, left ventricular diastolic dysfunction, LV-EF, left ventricular ejection fraction, MRI, magnetic resonance imaging, PVI, pulmonary vein isolation, S, mean deviation, SD, standard deviation

## Abstract

**Background:**

Left atrium (LA) remodeling is associated with atrial fibrillation (AF) and reduced success after AF ablation, but its relation with low-voltage areas (LVA) is not known. This study aimed to evaluate the relation between regional LA changes and LVAs in AF patients.

**Methods:**

Pre-interventional CT data of patients (n = 24) with LA-LVA (<0.5 mV) in voltage mapping after AF ablation were analyzed (Surgery Explorer, QuantMD LLC). To quantify asymmetry (ASI = LA-A/LAV) a cutting plane parallel to the rear wall and along the pulmonary veins divided the LA-volume (LAV) into anterior (LA-A) and posterior parts. To quantify sphericity (LAS = 1-R/S), a patient-specific best-fit LA sphere was created. The average radius (R) and the mean deviation (S) from this sphere were calculated. The average local deviation (D) was measured for the roof, posterior, septum, inferior septum, inferior-posterior and lateral walls.

**Results:**

The roof, posterior and septal regions had negative local deviations. There was a correlation between roof and septum (r = 0.42, p = 0.04), lateral and inferior-posterior (r = 0.48, p = 0.02) as well as posterior and inferior-septal deviations (r = −0.41, p = 0.046). ASI correlated with septum deformation (r = −0.43, p = 0.04). LAS correlated with dilatation (LAV, r = 0.49, p = 0.02), roof (r = 0.52, p = 0.009) and posterior deformation (r = −0.56, p = 0.005). Extended LVA correlated with local deformation of all LA walls, except the roof and the septum. LVA association with LAV, ASI and LAS did not reach statistical significance.

**Conclusion:**

Extended LVA correlates with local wall deformations better than other remodeling surrogates. Therefore, their calculation could help predict LVA presence and deserve further evaluation in clinical studies.

## 1 Introduction

Atrial fibrillation (AF) is associated with left atrial (LA) remodeling, characterized not only by dilatation but also by changes of LA symmetry. This is particularly true for greater LAs, when due to anatomical constrictions LA extension occurs non-uniformly. This asymmetric LA dilatation is a predictor of poor outcome after catheter ablation [1], [2], [3].

Extended low voltage areas (LVAs) as seen during intra-procedural mapping have been associated with worse outcomes and may reflect the need for further ablation [4]. While patients without AF substrate could benefit the most from a simple elimination of AF triggers by pulmonary vein isolation (PVI), some patients with LVAs may require modification of AF substrate to avoid recurrence. Therefore, the presence or extent of LA substrate as well as its localization can have a profound clinical impact on managing AF patients.

LA remodeling could provide pre-procedural information about AF substrate and help plan the procedure. The relationship of LA remodeling with LVAs though has not been adequately examined yet. This study aimed to use a new software suite and evaluate the relation between regional LA changes and LVAs in AF patients using novel geometry metrics.

## 2 Methods

### 2.1 Patients

We prospectively studied a total of 24 patients that underwent catheter ablation for symptomatic AF. All patients had a pre-procedural computed tomography (CT) for accurate depiction of LA and all patients had LVAs in the voltage mapping during sinus rhythm at the end of the procedure (selection criteria). Exclusion criteria were previous ablation for any arrhythmia or previous heart surgery, impaired left ventricular ejection fraction (LV-EF), severe valvular disorders, pacemaker stimulation and age <18 or >80 years. All patients gave written informed consent, the institutional committee approved the study and data were collected in accordance with the Declaration of Helsinki.

### 2.2 Echocardiography

Transthoracic and trans-esophageal echocardiography was performed (2 ± 1 days) before the procedure and intracardiac thrombi were ruled out. Images were acquired with the patients in the left lateral decubitus position using a commercially available system (Vivid-9 General Electric Vingmed, Milwaukee, WI, USA). Image acquisition was performed in the standard parasternal and apical views. Standard M-mode and 2D images, including color Doppler data from 3 consecutive heartbeats, were obtained according to current guidelines [5].

### 2.3 Computed tomography and shape analysis

Cardiac-CT was performed with a multidetector 64-row helical system (Brilliance 64, Philips Medical Systems, Best, The Netherlands) with the following parameters: 70–120 KV, 850 mAs, 0.6 mm beam collimation, 0.625–1.25 mm thickness and 20–30 cm field-of-view. During an end-inspiratory breath-hold of 20 s, and following a bolus-chase injection (20 mL, 5 mL/s), 90 mL of an contrast medium (Ultravist 370, Bayer Vital, Cologne, Germany) was administered. End-systolic imaging data were used for 3D reconstruction (EnSite Verismo, SJM, MN). After exclusion of the appendage (LAA) and the pulmonary veins (PV), the left atrial volume (LAV) was divided by a cutting plane, between the PV ostia and the LAA and parallel to the posterior wall, into an anterior (LA-A) and a posterior (LA-P) part. The ratio LA-A/LAV was defined as an index of asymmetry (ASI).

**Fig. 1:**
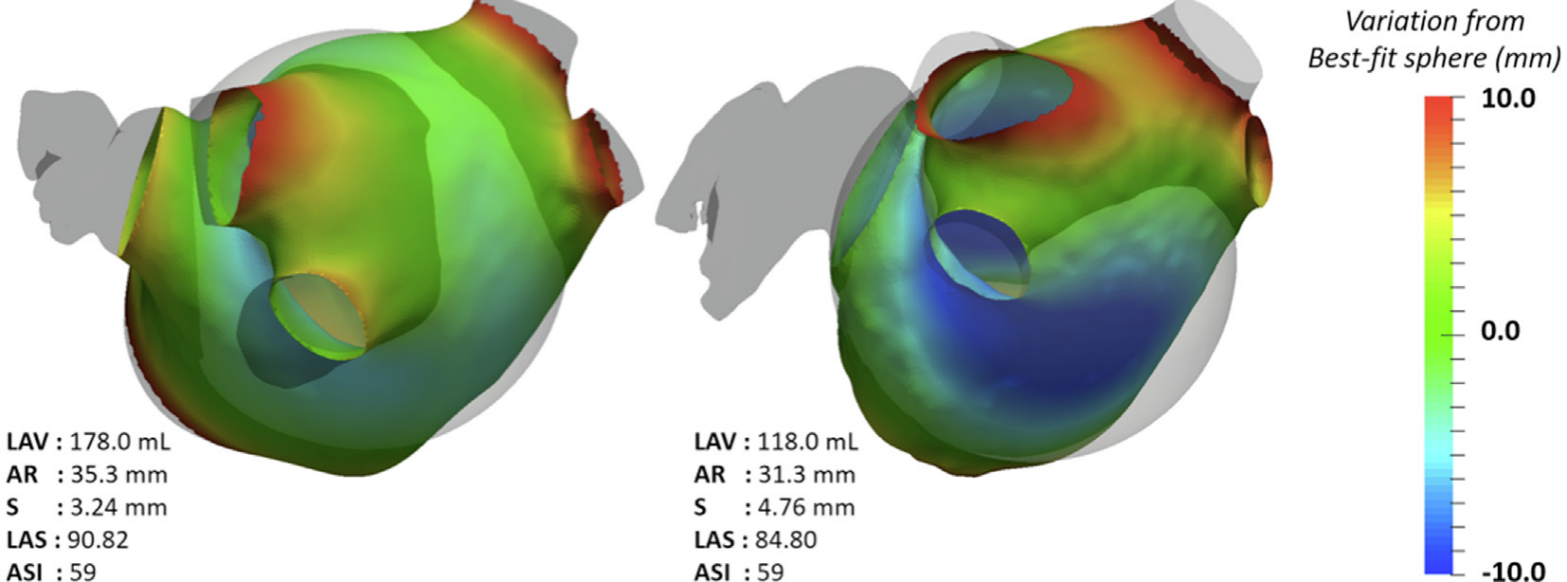
The average radius (AR) and the mean deviation (S) from a best-fit sphere in two patient-specific LA models, using iterative closest point registration. Regional deviation from the best-fit sphere is illustrated as a color-map, plotted in LA surface with blue for concave and red for convex areas. Regions >10 mm from the fitted spherical surface were excluded. (For interpretation of the references to color in this figure legend, the reader is referred to the web version of this article.)

Additional analysis was performed using a novel Visualization Tool Kit of special software, designed to quantify shape, sphericity (LAS) and regional deformations (Surgery Explorer, Quant MD LLC). DICOM images were segmented in 3D to extract the LA surface and an optimal sphere was fitted on a patient specific basis, using an iterative closest point (ICP) registration tool (Fig. 1). The ICP algorithm matched the closest points between a fixed point dataset (patient’s anatomy) and a floating point dataset (optimal sphere) using a similarity transformation (i.e. rigid body rotation, translation and scaling) as previously described [6]. Regions >10 mm from the fitted spherical surface (PVs, LAA) were excluded. The average radius (AR) and deviation (S) from the sphere were used to compute LAS (=1-S/AR) [2]. In other words, the software calculated a signed point-to-surface regional distance metric from the best-fit sphere to each patient-specific LA on the trajectory of a radius crossing the center of the sphere.

**Fig. 2:**
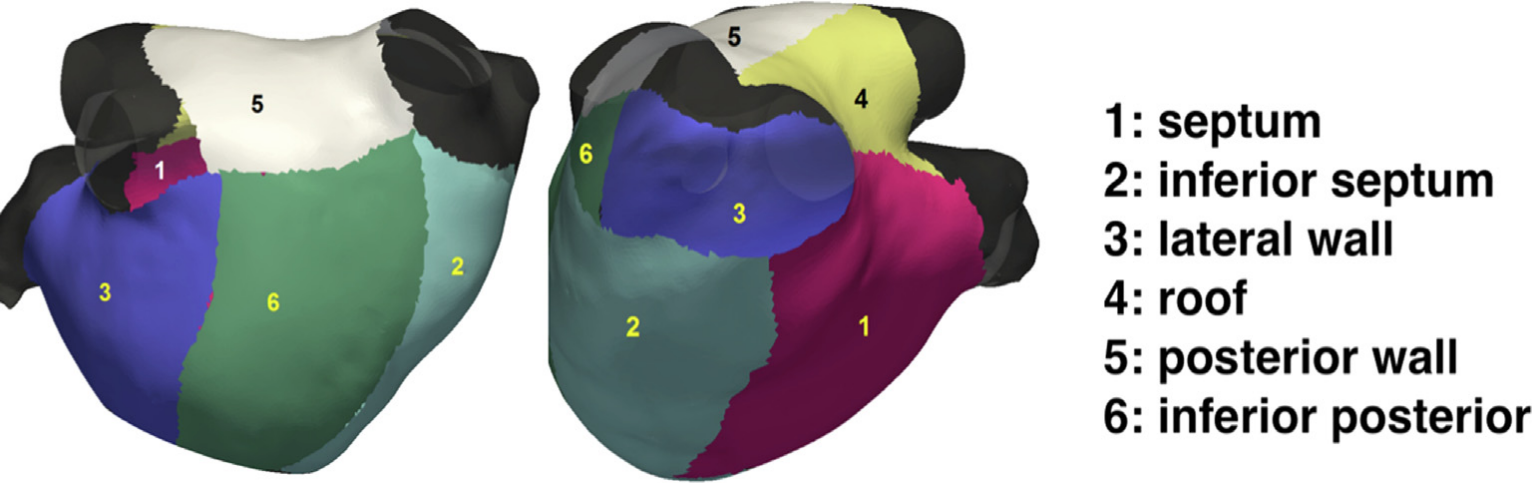
Illustration of regional partitioning of the patient-specific LA surface.

Next, each LA was partitioned into six surface segments, visualizing the inferior-posterior wall, inferior septum, anterior septum, roof, posterior wall and lateral wall segments (Fig. 2). The mean proximity of these segments from their respective closest locations on their best-fit spheres were recorded as a regional metric of segmental sphericity, named as average wall deviation (D).

Image analysis was performed offline by an experienced observer blinded to the results of the procedure and the patient’s characteristics. Initial measurements of 6 patients were repeated 4 weeks later by the same investigator and a second independent reviewer in a blinded fashion.

### 2.4 Mapping and ablation procedure

Catheter mapping and ablation was performed under sedation as previously described [4]. Transseptal access and catheter navigation were performed with a steerable sheath (Agilis, St. Jude Medical, St. Paul, MN, USA) and electroanatomic mapping systems (EnSite™ NavX™, St. Jude Medical; or CARTO™, Biosense Webster, Diamond Bar, CA, USA), after integration of CT image datasets. All patients received circumferential ablation lines around the ipsilateral pulmonary veins (irrigated tip catheter, temperature of ≤48 °C, power of 30–45 W). After restoration of sinus rhythm, complete PV isolation (PVI) was verified with a multipolar circular mapping catheter (Inquiry Optima or Reflexion Spiral; St. Jude Medical or Lasso; Biosense-Webster) and then a detailed bipolar LA voltage map was acquired. Additional points were acquired with a force-sensing catheter to ensure adequate contact and substrate modification was performed as needed. Approximately 130–230 evenly distributed mapping points were systematically acquired with an interpolation threshold of 5 mm or less. The ablation catheter was additionally used to create high-density maps in difficult to reach areas, using different catheter angulations and maneuvers and providing sufficient wall contact force (>5 gr). In accordance with previous studies [7], [8], [9], [10], LVAs were defined as sites of ≥3 adjacent points <0.5 mV in the above-mentioned LA segments.

### 2.5 Statistics

**Fig. 3:**
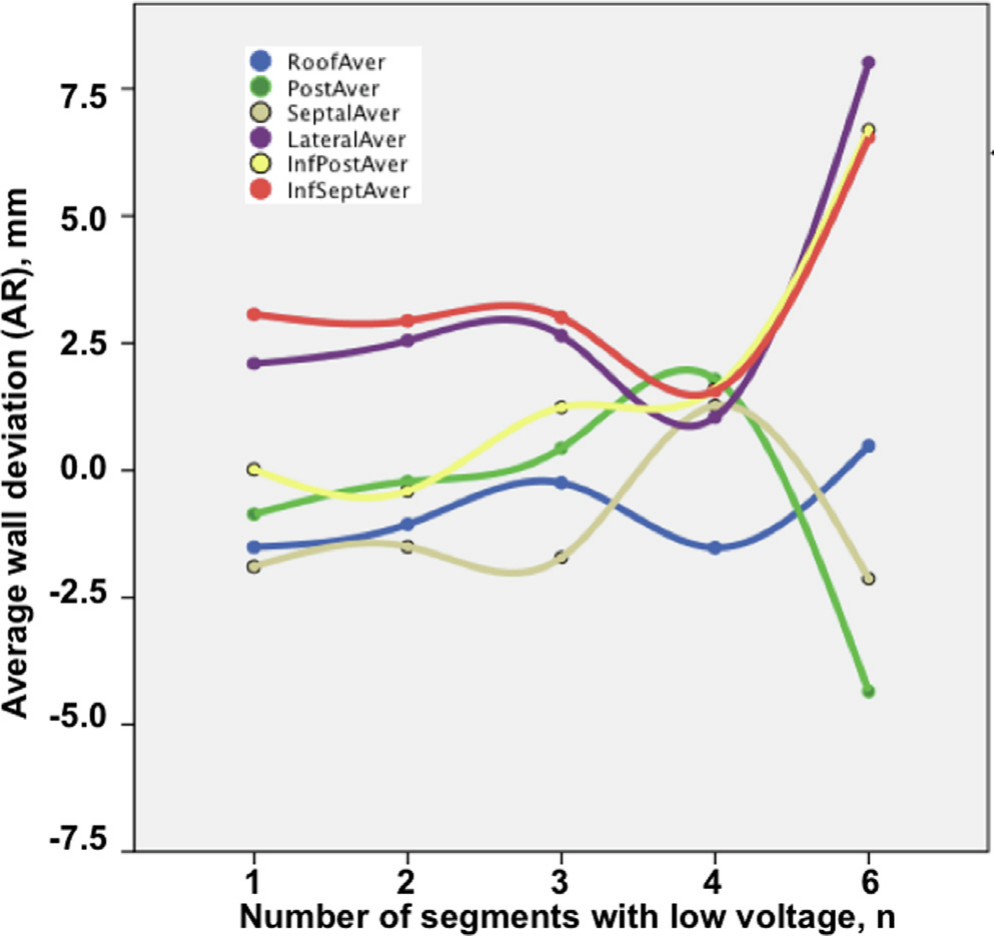
Extended left atrial scar (more segments with low voltage) was associated with increased outward deformation of the lateral, inferior posterior and inferior septal LA areas as well as increased inward deformation of the posterior wall.

Continuous variables are expressed as mean and standard deviation (SD) when normally distributed (positive Kolmogorov-Smirnoff test) or as median and interquartile range (IQR). Categorical variables are reported as frequencies and percentage. Parametric variables were compered by means of paired Student’s *t*-test and non-parametric variables by Wilcoxon-test or chi-square test. Signed Spearman rank correlations between wall deformation metrics and LAV as well as global LAS were evaluated to derive an association of regional and global features of atrial shape. The association between the number of regions with LVA and the magnitude of regional wall deformation was depicted on a graph (Fig. 3). Extended LVA (all 6 segments) correlation with wall deformation was tested with the Spearman rank test and differences were compared with Student’s *t*-test. Intra-observer and inter-observer variability was expressed with Pearson’s correlation coefficient (r). A two-tailed P-value less than 0.05 was considered statistically significant. Analysis was performed with SPSS v20.0 (SPSS Inc., Chicago, USA).

## 3 Results

### 3.1 Patient characteristics

Patients had a mean age of 71 ± 8 years, CHADS-VASc of 2.8 ± 1.5, LAV 155 ± 35 mL, ASI 67 ± 5% and LAS of 82 ± 6% (Table 1). The intra- und inter-observer correlation of LA measurements (LAV, LA-A, LA-P, ASI and regional local wall deviations) was found to have coefficients of ≥88% [1], [3]. The roof, posterior and septal regions had negative, whereas other regions had positive local deviations.

**Table 1:** Clinical characteristics and LA measurements (global and regional) for AF patients with low voltage areas during sinus rhythm.

**Baseline characteristics**		**LA measurements**	
Age, years	66 ± 8	**Echocardiography**	
BMI, kg/qm	30 ± 5	LV ejection fraction, mm	57 ± 11
Height, cm	169 ± 8	LV diameter, mm	45 ± 7
Female, n (%)	14 (58)	LA diameter, mm	45 ± 6
Persistent AF, n (%)	19 (79)	**Computed tomography**	
Heart failure, n (%)	3 (13)	LA volume (LAV), ml	155 ± 35
Coronary disease, n (%)	4 (17)	Anterior LA volume, ml	104 ± 23
Stroke, n (%)	1 (4)	Posterior LA volume, ml	52 ± 16
Diabetes, n (%)	6 (25)	Asymmetry index (ASI), %	67 ± 5
Hypertension, n (%)	21 (88)	LA sphericity (LAS) %	82 ± 6
Hyperlipidemia, n (%)	12 (35)	Average radius (R), mm	32 ± 4
CHA_2_DS_2_-VASc score, n	2.8 ± 1.5	Mean deviation (S), mm	6 ± 2
**Low voltage areas**		**Local wall deviation**	
– Roof, n (%)	16 (67)	– Roof (D), mm	−0.9 ± 3
– Posterior, n (%)	13 (54)	– Posterior (D), mm	−0.6 ± 2
– Septum, n (%)	15 (63)	– Septum (D), mm	−1.6 ± 2
– Inferior septum, n (%)	2 (8)	– Inferior septum (D), mm	3.2 ± 3
– Inferior posterior, n (%)	7 (29)	– Inferior posterior (D), mm	0.8 ± 2
– Lateral, n (%)	2 (8)	– Lateral (D), mm	2.8 ± 3

LA = left atrial, LV = left ventricular.

### 3.2 LA remodeling and low-voltage

There was a correlation between roof and septum (r = 0.42, p = 0.04), lateral and inferior-posterior walls (r = 0.48, p = 0.02) as well as posterior and inferior-septal local deviations (r = −0.41, p = 0.046). Asymmetry (ASI) correlated with septum deformation (r = −0.43, p = 0.04). Sphericity (LAS) correlated with LA dilatation (LAV, r = 0.49, p = 0.02), roof (r = 0.52, p = 0.009) and posterior LA changes (r = −0.56, p = 0.005).

The number of LVA segments was associated with significant differences in local deformation of all LA walls (p < 0.05), except the roof (p = 0.69) and the septum (p = 0.67). LVA of lateral or inferior septal walls (n = 2) had similar effects whereas inferior posterior LVA (n = 7) resulted only in local changes. Most patients had one (n = 8, 33%) or two (n = 8, 33%) but some had 3 (n = 5, 21%), 4 (n = 1, 4%) or 6 (n = 2, 8%) segments of LVA. Most commonly LVA was seen on the roof (n = 16, 67%), the septal (n = 15, 62%) and posterior (n = 13, 54%) wall and less frequently on the inferior posterior (n = 7, 29%), lateral (n = 2, 8%) and inferior septal wall (n = 2, 8%). Extended LA scar (6 LVA segments) was associated with increased outward deformation of the lateral (r = 0.37, p = 0.045), inferior posterior (r = 0.48, p = 0.018) and inferior septal (r = 0.37, p = 0.045) LA areas as well as increased inward deformation of the posterior wall (r = −0.41, p = 0.044, Fig. 3). Patients with extended LVA had significantly higher average deviations of the lateral (8.0 ± 4.9 vs. 2.4 ± 2.6, p = 0.01), inferior-posterior (6.7 ± 1.7 vs. 0.1 ± 1.7, p < 0.001) and inferior septal walls (6.5 ± 0.3 vs. 3.0 ± 2.6, p < 0.001) as well as negative deformation of the posterior wall (-4.3 ± 1.7 vs. −0.2 ± 1.5, p = 0.008) than patients with less LVA extent (1–3 segments). LVA association with LAV, ASI & LAS did not reach statistical significance.

## 4 Discussion

### 4.1 Main findings

We created a novel descriptive metric of atrial wall deformation measured by a special software and compared this with current surrogates of atrial remodeling for their association with the presence of low-voltage areas (LVA) in patients presenting for an AF ablation. We found that the local deviation (from a best-fit sphere) of the atrial walls correlates with extended LVA better than other remodeling surrogates, such as asymmetry of sphericity. To the best of our knowledge, this is the first study that applies a new metric of regional shape changes and shows association with electrophysiologic characteristics of the underlying tissue. Therefore, calculation or these changes could help predict LVA presence and deserve further evaluation in clinical studies.

### 4.2 Atrial remodeling and clinical implications

Historically, atrial remodeling has been mostly studied as LA enlargement that correlated with clinical outcomes such as rhythm stability, thromboembolic risk and mortality [1], [2], [11]. Atrial dilation is closely related to AF risk, and atrial stretch is known to promote AF [12]. However, the physical constraints of the spine and the sternum [13], the changing tissue characteristics [14] and the pathophysiologic mechanisms result in a non-uniform enlargement that is more prominent for the anterior LA part [1]. New surrogates of remodeling, like the asymmetry and sphericity index, have been developed to better reflect these changes, but hitherto no studies have examined the regional wall deformation.

In this proof of concept study, we developed a new Visualization Tool Kit designed to quantify shape and provide novel metrics like regional deformations. We used this tool in a series of patients carefully examined for low-voltage areas and found that atrial wall changes correlate with the extent of fibrotic tissue. This probably represents the cumulative effect of wall stress, atrial expansion and anatomical constrictions that add to the geometrical dispersion of refractoriness and the perpetuation of spiral fibrillatory waves [15], [16]. Thus geometrical variance could be associated with source-load mismatch and regional anisotropic conduction properties. These results supplement previously published data showing that asymmetry increases as the LA volume expands, especially at the initial (paroxysmal) stages of AF, when remodeling is primary driven by dilatation [3]. This adds up to the studies that examine the impact of LA shape and fibrosis on atrial arrhythmogenesis [16], [17], [18], [19], and emphasizes the importance of patient-specific anatomical information in the context of AF.

Despite the previously reported association of asymmetry or sphericity index with clinical outcomes, these surrogates of remodeling did not correlate with the extent of scar tissue (e.g. LVAs). This could be explained by the small number of the patients, most of which had paroxysmal AF, or by the fact that local changes may better represent the extent of fibrosis. The present findings though suggest that regional wall deformation could provide incremental information that could help plan an ablation strategy using simple one-shot devices or radiofrequency substrate-targeting strategies for advanced AF stages.

### 4.3 Limitations

This study has several limitations. First, the elaborate analysis of novel remodeling metrics, requiring manual segmentation, has limited the number of analyzed patients. The segmentations were reproducible but time-intensive, complex and cumbersome. Thus only 24 patients were used for this proof of concept study and association with procedural outcomes or comparison to a control group was not possible. Previous studies though have revealed that LA remodeling in AF patients is associated with increased volumes and asymmetry compared to healthy controls [1]. Automatic segmentation and analysis of atrial wall deformation will be soon available allowing for wider application of these metrics. Imaging with computed tomography required radiation and contrast dye exposure that prohibited follow-up studies. Certainly, MRI based imaging may have provided more insights about shape changes and fibrosis or repeat studies [20]. MRI is currently not widely or readily available, but shape analysis could be easily applied on MRI or 3D echocardiography data [21]. PV ostial regions could confound the results, but this area was no assessed for LVAs after PVI. Finally, this study included only patients with present substrate (LVA) and did not include reference values from AF patients without LVAs. However, correlation between local wall changes and LVA extent reached statistical significance. Thus, even with the above limitations, the findings of this study are hypothesis generating and deserve further evaluation.

### 4.4 Conclusions

In this proof of concept study a novel measurement of local wall deformation correlated with extended LVA better than other remodeling surrogates (LAV, ASI or LAS). Therefore, their calculation could help predict LVA presence and deserves further evaluation in clinical studies.

## Data availability

Data are available upon reasonable request from the corresponding author.

## Declaration of Competing Interest

The authors report no relationships that could be construed as a conflict of interest.
